# Data Stream Processing for Packet-Level Analytics †

**DOI:** 10.3390/s21051735

**Published:** 2021-03-03

**Authors:** Alessandra Fais, Giuseppe Lettieri, Gregorio Procissi, Stefano Giordano, Francesco Oppedisano

**Affiliations:** 1 Dipartimento di Ingegneria dell’Informazione, Università di Pisa, 56122 Pisa, Italy; giuseppe.lettieri@unipi.it (G.L.); gregorio.procissi@unipi.it (G.P.); stefano.giordano@unipi.it (S.G.); 2 NetResults S.r.l., 56121 Pisa, Italy; oppedisano@netresults.it

**Keywords:** software defined networking, packet-level analysis, data stream processing, multicore programming

## Abstract

One of the most challenging tasks for network operators is implementing accurate per-packet monitoring, looking for signs of performance degradation, security threats, and so on. Upon critical event detection, corrective actions must be taken to keep the network running smoothly. Implementing this mechanism requires the analysis of packet streams in a real-time (or close to) fashion. In a softwarized network context, Stream Processing Systems (SPSs) can be adopted for this purpose. Recent solutions based on traditional SPSs, such as Storm and Flink, can support the definition of general complex queries, but they show poor performance at scale. To handle input data rates in the order of gigabits per seconds, programmable switch platforms are typically used, although they offer limited expressiveness. With the proposed approach, we intend to offer high performance and expressive power in a unified framework by solely relying on SPSs for multicores. Captured packets are translated into a proper tuple format, and network monitoring queries are applied to tuple streams. Packet analysis tasks are expressed as streaming pipelines, running on general-purpose programmable network devices, and a second stage of elaboration can process aggregated statistics from different devices. Experiments carried out with an example monitoring application show that the system is able to handle realistic traffic at a 10 Gb/s speed. The same application scales almost up to 20 Gb/s speed thanks to the simple optimizations of the underlying framework. Hence, the approach proves to be viable and calls for the investigation of more extensive optimizations to support more complex elaborations and higher data rates.

## 1. Introduction

In recent years, we have witnessed a boost in the research activity revolving around Software Defined Networks (SDNs) and Network Function Virtualization (NFV), supported by steady technological advancement. Today’s network infrastructures (*i*) are designed to be shared among a plethora of services; (*ii*) they must be able to rapidly change their configuration according to newly detected alterations in the network status; (*iii*) they mostly rely on programmable network equipment, whose behavior can be configured in software. If a prompt re-configuration of the network does not occur when needed, two major risks are that (*i*) the Quality of Service (QoS) requirements for each running service may not be satisfied any more and the (*ii*) security may be severely impaired. Therefore, effective re-configuration capabilities, and the ability of implementing efficient continuous monitoring features are two aspects of utmost importance for network operators. Indeed, signs of *performance degradation* or *security threats* must be tracked as soon as possible (in real time, or very close to it) so as not to undermine the execution of the above running services. Service disruption, failures, long down-times, and so on are not only harmful events per se but may also result in huge economic losses for service providers (and network operators as a consequence) if they are not dealt with in a prompt way. Preserving the “good health” of the infrastructure and keeping it reliable is therefore a fundamental goal.

It is possible to identify two main categories of solutions designed to meet these requirements:

- *Reactive* approaches detect and respond to performance and security alterations. To be effective, they need to identify in real time any alarming alterations caused by extracting useful information from the analyzed data. As a consequence, the configuration of the network must be rapidly changed to correct the detected problem(s).
- *Proactive* techniques try to predict possible sources of performance degradation or security attacks. When signals of a potential issue are detected, the network configuration is modified to prevent the identified problem from occurring. Solutions belonging to the Machine Learning and Artificial Intelligence (ML/AI) domains are good candidates to implement this kind of approach.

In both cases, the availability of tools for the high-performance continuous monitoring of network traffic parameters through packet-level analysis is crucial. In this area, in the last few years there have been significant research efforts focused on finding more effective ways of implementing network telemetry and monitoring functionalities. On one hand, there is the need to achieve high *performance* in terms of both throughput and latency. Therefore, proper processing solutions capable of handling very high input rates (in the order of tens of gigabits per second) must be devised to offer new tools supporting the implementation of general-purpose real-time computations over streaming data in modern networks. On the other hand, the degree of *expressiveness* that a tool is able to offer can greatly influence its audience. The higher the expressive power and the Application Programming Interface (API) abstraction level, the wider the community of people that could benefit from the adoption of the proposed solution. The aim is to let complex and general streaming computations be designed in a simplified way by end users who implement network monitoring applications using the framework.

**Figure 1:**
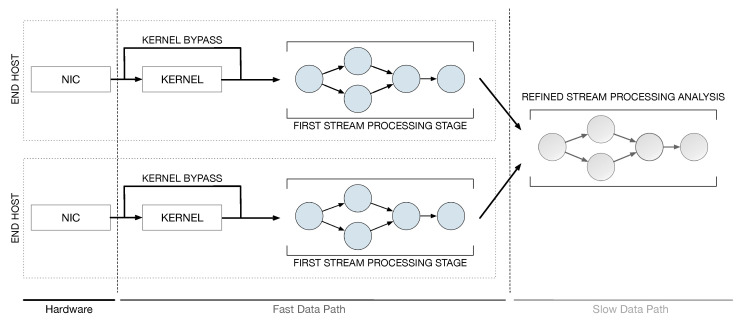
High-level view of the framework architecture.

This work addresses the issue of implementing fast and scalable applications for real-time continuous network monitoring on end-host machines. To this end, we propose a framework that combines the power of software accelerated packet capture engines with the expressiveness of the Data Stream Processing (DaSP) programming paradigm. Scalability is further improved by implementing the entire processing according to a two-layer structure. In the context of this work, we will borrow the terms fast data path and slow data path to identify the two processing phases. The term fast data path identifies the first layer of processing on the packet itself upon its reception from the Network Interface Card (NIC) in the end host, while a slow data path identifies an optional processing level that can be implemented to produce a visual representation of the statistics and aggregated data produced by the fast data path. Figure 1 offers a high-level view of the proposed framework architecture. The processing pertaining to the slow data path can be either executed in the same end-host machine or in a dedicated one, that can possibly elaborate on the data produced by multiple end hosts. The general idea can be summarized in the following:

- In the fast data path, queries that are continuously executed on packet streams at line rate can be implemented by DaSP frameworks, as they specifically address real-time processing of data in the form of *streams*.
- So far, DaSP frameworks have been typically applied for developing streaming applications in traditional distributed environments (i.e., homogeneous clusters) and they achieve platform independence by relying on the Java Virtual Machine (JVM). As such, their performance does not quite match the level required to handle packets over the fast data path of network applications. Hence, in this work we specifically selected an innovative C++-based streaming library targeting multicores (i.e., WindFlow [1,2]) and applied proper optimizations to extend its application domain to packet-level analysis. In addition, suitable extensions for accommodating new accelerated data sources have been designed and provided.
- In case the elaboration produced in the fast data path is not sufficient and need to be refined by a second level of logic, the aggregated results of the first stage may be forwarded to a second processing tier (slow data path) in which the queries executed on streams of pre-digested statistics can be implemented with any DaSP framework. This is possible thanks to the lower data rate at which partial results are received from the first processing stage. In fact, the performance achievable by traditional DaSP frameworks are typically sufficient at this stage of the computation.

In our previous work [3], we first presented the main perspectives and challenges of the adoption of Data Stream Processing in network application scenarios. This work extends [3] in two main directions: (*i*) we present the design of a higher-level framework to improve the performance of packet-level analysis applications by taking advantage of suitable stream processing systems in conjunction with fast packet capturing tools; (*ii*) the concept of *a multi-layer stream processing model* is here applied to the implementation of a first example application that is used as a benchmark tool to prove the viability of the stream processing approach.

The rest of the paper is organized as follows. Section 2 presents the state-of-the-art research on network programmability by summarizing the main programming abstractions, high-level frameworks, and high-speed packet-capturing tools. After that, an extensive discussion is dedicated to the Data Stream Processing computational model in Section 3. Section 4 details the structure of the proposed framework, and this is followed by the description of a first example application in Section 5. The results of the experimental evaluation are presented and discussed in Section 6. Finally, Section 7 points out the most promising research directions and draws the conclusions of the paper.

## 2. Background

The natural evolution of SDNs and NFV is represented by the *softwarization* of networks. Specialized hardware is replaced by general-purpose devices and network functions are implemented as software applications that can be instantiated through the network control plane. Therefore, the controller enforces the operations of programmable nodes on the data plane and is in charge of a second layer of processing applied to the early stage output received from the data plane. This novel processing paradigm raises a number of issues from different points of view:

- the need for the availability of proper APIs and programming abstractions to allow the control plane to configure and instruct the data plane processing nodes;
- the data plane itself must be able to perform early-stage data elaboration at (or nearly at) the network line rate;
- the control plane must be able to cope with the possibly significant computation burden imposed by different use-cases—for example, a system for Deep Packet Inspection (DPI), or, even more complex, Security Information and Event Management (SIEM).

### 2.1. Programmable Abstractions

The very first approach to network programmability in SDNs came from the switching domain, with the simple *match-and-action* abstraction brought by OpenFlow [4]. While still quite limited, the OpenFlow abstraction was successful in forcing manufacturers and vendors to start working on the real-world deployment of fully programmable networks. In the last few years, P4 [5] has widely extended the concept of node programmability becoming the new *de facto* standard language for programmable switches with the Protocol Independent Switch Architecture (PISA), such as [6]. Molnar et al. in [7] implement a novel switch architecture based on Intel DPDK [8]. Their solution is capable of compiling any OpenFlow pipeline into machine code, which can be used as forwarding path. The aim is to improve the performance of the OpenFlow pipeline execution, and the solution has been experimentally compared against Open vSwitch [9]. Sun et al. in [10] propose the implementation of a protocol independent dataplane to support special-purpose protocols that are not yet standardized in OpenFlow. As in Molnar et al. [7], this solution is also based on Intel DPDK. Moreover it proposes a flow table management scheme to further improve the packet forwarding throughput of the software-based Protocol Oblivious Forwarding (POF) switch. Other abstractions originating from the academic community [11,12,13] address the management of stateful processing on hardware platforms through the use of eXtended Finite State Machines (XFSM). While the above approaches target programmable hardware nodes, other programming abstractions for software solutions have been made available in the meantime. Enif-lang [14] extends the works in [15,16], providing a tool for programming generic network functions on the Linux Operating System (OS). Program safety and correctness are enforced thanks to its strongly typed nature, inherited from the underlying used Haskell functional language. Other approaches based on imperative languages [17,18,19] provide solutions for aspects such as describing the data plane logic of SDNs [17] or configuring I/O paths to applications through the OS [19]. Cisco’s Vector Packet Processing (VPP) [20] provides a high-performance packet-processing stack that can run on commodity CPUs for implementing production quality switch/router functionalities. Recently, the extended Berkeley Packet Filter (eBPF) [21] proposes itself as a promising approach, natively supported in the Linux OS kernel. This solution is gaining more and more popularity thanks to the ability to offer high flexibility and high performance over low-cost platforms.

### 2.2. Software Accelerated Packet Handling

The technological maturity reached by Commodity Off The Shelf (COTS) hardware platforms makes general-purpose servers reasonable candidates for implementing real network nodes. As a result, a considerable number of approaches have been proposed in the literature for efficient packet handling at sustained rates. A complete review on the limits of possible general-purpose solutions for the capture level can be found in [22,23,24]. Software-based solutions for high-performance packet processing generally exploit *multi-core parallelism*, *kernel bypass* techniques, and *offloading* features in order to keep up with high packet rates. Often in this scenario, network applications performing packet processing are given direct access and control over the network hardware, and some CPU cores can be dedicated exclusively to performing computation on the received traffic. Representative examples of frameworks implementing this approach are the Data Plane Development Kit (DPDK) [8]; Netmap [25]; PF_RING [26]—and its Zero Copy (ZC) [27] version; and PFQ [16]. PF_RING [26] was historically the first accelerated tool for packet capturing over 1 Gbps links. Later on, PF_RING ZC [27] and Netmap significantly pushed forward the performance of capture engines up to multi-gigabit line rate by memory mapping the ring descriptors of Network Interface Cards (NICs) at the user space. Intel’s DPDK and PFQ added room for packet processing beside high-speed capture rates. The former is based on the concept of bypassing the OS, while the latter is a software acceleration engine built upon standard network device drivers that primarily targets packet fan-out.

Therefore, two main points are exploited by these capturing solutions:

- the overhead related to the interface positioned between the OS kernel and the userspace can be removed by fully bypassing it;
- copies of data between userspace and kernel memory can be avoided, getting rid of the other important source of overhead.

This is without a doubt an effective way to achieve higher performances on COTS hardware [24]. However, some drawbacks derive from kernel bypassing. Among others, these include the complex integration with the rest of the system and the need to re-implement functionalities otherwise provided in the network stack and security. In order to address these negative aspects, a new approach for fast programmable packet processing integrated in the operating system networking stack has recently been proposed. The eXpress Data Path (XDP) [28] cooperates with the networking stack by providing a safe execution environment—a VM running eBPF code—which offers custom packet processing capabilities in the device driver context.

As for hardware-based solutions, software-based approaches offer offloading features to move a portion of—or all of—the workload to the NIC, saving CPU cycles [24]. Offloading is supported by several software-based solutions—among others, XDP/eBPF. This allows the programmer to explicitly delegate a partition of the processing to a co-processor—FPGA, GPU, SmartNIC—if it is available, while keeping the CPU busy with the execution of the other portion of the computation.

It is also worth mentioning that the Linux kernel itself has significantly improved its native capture performance with the adoption of the TPACKET (version 3) socket [29], and that the pcap library has also been extended [30] to support packet fan-out and enable multi-core processing.

### 2.3. Higher–Level Frameworks

The availability of programmable abstractions and software accelerated frameworks provides the necessary substrate for fast end efficient data-plane programming on both specialized (PISA switches) and general-purpose hardware platforms. However, all such tools provide low-level abstractions and still require highly skilled end users to properly use them. Hence, recent works have addressed the proposal of higher level frameworks with improved expressiveness to ease the life of network developers. In-Band Network Telemetry (INT) applications execute on top of PISA switches and give access to switch-internal state parameters of a switch, such as queue size, link utilization, and queueing latency. Kim et al. in [31] use P4 to define the switch match-action pipeline processing, and each switch in the network uses INT to periodically push telemetry packets containing values such as switch ID and queueing time to an end host. Tang et al. in [32] propose and design a runtime programmable selecting INT scheme which is based on POF. First, they prototyped their design by extending the Open vSwitch platform to process packets in a protocol-agnostic way. Second, they implemented, on top of this new software switch, a new runtime-programmable network monitoring system that supports changing locations to collect INT information, new definitions of INT data types, and so on, in runtime. In addition, a data analyzer module (implemented as a hardware-based commercial product) can capture, parse, and store INT data carried by packets. UnivMon [33] implements a generic monitoring primitive on switches/routers, providing an RISC-type approach to measurement. The idea is to have a single universal sketch designed to support a range of monitoring functions. A slightly different approach is to have a specialized sketch per measurement task. This is the idea implemented by OpenSketch [34], which offers a library of predefined functions in hardware that can be selected and combined by the controller to implement different tasks. Among these building blocks we can find count-min sketch, hash table, Bloom filter, and others that simplify the implementation of tasks. However, to monitor different tasks, many concurrent sketch instances need to be executed, one for each task, requiring an increased amount of resources. Since computational resources on switches are limited, only a restricted set of simple metrics can be monitored at any time. Another work that deserves mention is the one of Narayana et al. [35], which introduces an SQL-like declarative query language for defining a range of network performance queries (e.g., select high-latency packets, compute aggregated per-flow statistics) on the switch. However, to support the execution of more complex queries, there is the need to go beyond the domain of relational algebra. Indeed, complex applications such as frequent or rare pattern-mining on streams (useful for detecting security issues) [36,37,38] are not expressible in terms of relational algebra operators on tables (e.g., select, join), as well as operations involving the processing of packet payloads.

Higher expressive power is typically offered by solutions based on stream processing techniques on general-purpose CPUs. Works in Borders et al. [39] and Cranor et al. [40] offer SQL–like languages to implement monitoring queries. However, as stated before, many complex applications cannot be expressed as compositions of relational algebra operators. NetQRE [41] is a declarative language based on the usage of *regular expressions*. The defined monitoring tasks work on inputs which are streams of packets. A compiler generates efficient low-level imperative code to implement the specification, which is run on general-purpose CPUs. Packets from the network are processed in a streaming fashion and the produced output is a set of analysis results or actions taken to reconfigure the network. Tools for network security analytics such as [42,43] are based on the Apache Storm [44] stream processing system. These two works extend the range of monitoring tasks that can be implemented by reaching an expressive power similar to the one of mainstream stream processing frameworks (see Section 3). However, the throughput they are able to achieve is not sufficient with 10+ Gb/s traffic rates. Indeed, solutions based on state-of-the-art stream processing frameworks (Storm, Flink [45], Spark Streaming [46]) are typically deployed on distributed scenarios, and their performance results are achieved by scaling out the computation over a cluster of commodity machines. Therefore, the performance and scaling limitations of [42,43] come from overheads related to cluster coordination, in addition to the costs of packet capturing from the data plane and packet parsing before the analysis phase. In particular, the collection and parsing phases have a great impact on the throughput, since the stream processor processes all of the received packets. Moreover, the presence of the Java Virtual Machine, among other factors, makes these solutions ineffective in exploiting at best the computational resources offered by modern hardware in scale-up configurations (i.e., single machines with multi-core CPUs and co-processors such as GPUs and FPGAs) [47,48]. More details on this will be discussed in Section 3. A recent approach proposed with Sonata [49,50] stands in between the two families of solutions described above. The idea is to achieve both good performance and expressiveness by designing a unifying framework that takes advantage of PISA platforms and stream processors together. Monitoring queries can be expressed by means of a declarative interface, and they are partitioned across a stream processor and a switch. Higher packet rates are supported in this case, thanks to the reduction in the workload on the stream processor (implemented with Spark Streaming). This was a major performance limiting factor in solutions [40,41,42,43], where the stream processor has to process all of the incoming packets.

## 3. Data Stream Processing

Since the core of our proposal relies on the application of the Data Stream Processing paradigm in the design and development of network applications, this section elaborates upon the main principles and concepts behind this programming model and presents the main currently available frameworks.

**Figure 2:**
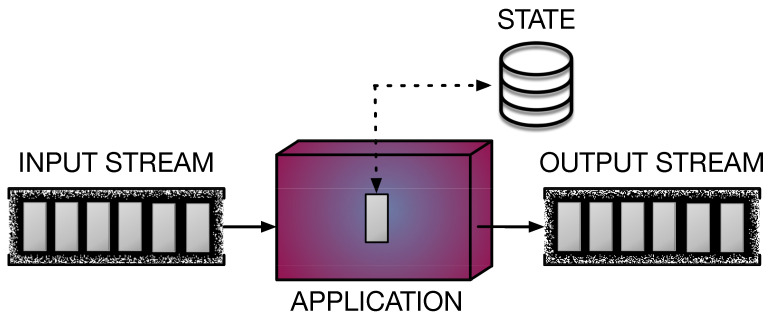
Stream processing application. Stream elements are represented in grey. The application receives a stream in input and performs some computation on each received item, producing a stream of results in output. Operators can be *stateless* or *stateful*, in case they maintain a state containing information on the data processed so far.

DaSP is a computational model that has gained more and more interest from the research community in recent years. Given the ever-increasing amount of data produced by a variety of devices (e.g., sensors of any kind) in the form of streams, DaSP proposed itself as a solution to meet the need for the continuous processing and real-time analysis of these streaming data. The adoption of DaSP is beneficial for several domains, such as networking, smart cities, smart mobility, and smart logistics, to name a few. Therefore, the widespread interest in developing new solutions for implementing fast computations over streams pushed the innovation in the DaSP research area. Different DaSP frameworks have been designed to offer suitable programming abstractions for implementing efficient applications processing streams in a simpler way. In general, such applications must be capable of performing real-time computations *on-the-fly*, as new data appear on the input streams. Operator parallelization and distribution are exploited in order to achieve the tight constraints imposed on throughput and latency performance parameters [51]. Figure 2 shows the general model of a streaming application.

*What is a stream?* In the DaSP context, the word *stream* identifies unbounded sequences of records of attributes in the form of key-value pairs, called *tuples*. Stream items continuously arrive at very high speed, and possibly with irregular and time-varying rates, and their significance decays over time. Implementing real-time computations over this kind of data imposes to find the correct strategies to deal with streams’ peculiarities, and this complexity needs to be managed in the streaming program.

*Why do we need the DaSP computational model?* In order to guarantee a real-time kind of processing, tight constraints must be placed in terms of *bandwidth* (output rate) and *latency* (response time). For this reason, new stream processing approaches have been proposed, as opposed to the more traditional *batch computing* model. The latter cannot be adopted to implement efficient real-time computations on streams, due to the impossibility of storing the whole data set (which is potentially infinite) before starting the computation. Indeed, this is the typical approach adopted to implement *offline computations*, after a first phase where large amount of data are collected over a period of time and stored (in databases or distributed file systems). Conversely, DaSP offers a way to implement *online computations* that are continuously executed as new data flows in the input stream, with no need to store any. There are situations in which we may need to maintain a small buffer (*state*) of the items that recently traversed the stream. In this cases, we can work on *windows*, where limited portions of the data set are stored. Windows are updated as the stream advances (e.g., moving windows) according to given semantics [52].

**Figure 3:**
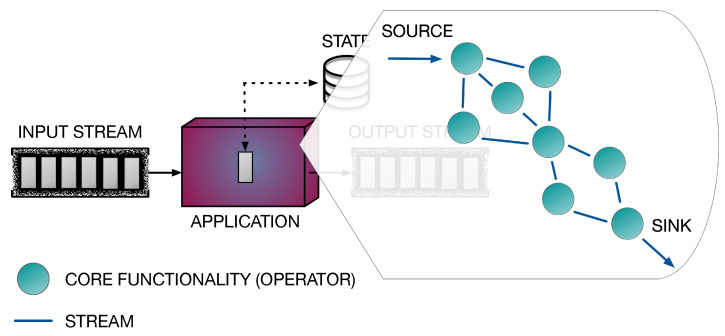
Data-flow graph representation of a stream processing application. The processing pipeline is defined by the operators (nodes) in the graph. Their business logic is executed on every stream item traversing the corresponding path in the graph. The application logic applied to each flowing element results from the execution of successive transformations in the graph.

*What is the role of parallelism?* Parallelism is exploited by DaSP frameworks to speed up the execution of streaming programs. The computational model adopted underlying is typically a data-flow graph. Therefore, a streaming application is modeled as a graph of core functionalities composed together to provide the complete computation. These *operators* or *transformations*—as the core functionalities are typically called in the DaSP context—correspond to the graph’s nodes. They are connected together by arcs, which model streams. In the end, arcs define the entire execution flow, from the *source* node to the final stage (i.e., the *sink*), through data dependencies. Figure 3 shows a possible data-flow graph representation of a streaming application.

*What are the main distinctive features of DaSP frameworks?* DaSP frameworks all share the aim of simplifying the development of efficient streaming applications. To this end, they typically provide suitable abstractions to mask the complexities related to stream handling (e.g., irregular arrival rates, decay of data significance over time) and parallelism management (e.g., communication patterns, synchronization problems). However, not all the frameworks provide the same level of abstraction. In fact, the main characterizing traits for a DaSP framework can be identified in the API exposed to the programmer and the runtime it is based on [53].

Mainstream solutions—also called Big Data Stream Processing (BDSP) frameworks [54]—such as Apache Storm [44], Flink [45], and Spark Streaming [46], target distributed systems of homogeneous machines (*clusters*). They typically rely on the Java Virtual Machine (JVM) processing environment and increase the application throughput as needed by scaling out the computation across multiple computing nodes in the cluster. Therefore, many data-flow graphs can be executed in parallel, and operators in each graph can be parallelized as well by exploiting stateless replication and stream partitioning [51]. The adoption of the JVM facilitates obtaining platform independence, at the price of processing overheads induced by factors such as garbage collection and data serialization and de-serialization. Thus, in these state-of-the-art solutions, the design of efficient data accesses and, as a consequence, the overall performance is limited by the presence of overheads related to the runtime and support to distributed systems. For these reasons, mainstream DaSP frameworks are not capable of efficiently exploiting the full hardware capabilities offered by single scale-up servers equipped with multi-core CPUs and co-processors (e.g., GPUs and FPGAs), as discussed in [47,48].

Alternative DaSP frameworks still offering high-level abstractions exist, such as the WindFlow [1] library. This parallel library is a tool to implement efficient stream processing targeting single multicore machines, with integrated support for GPUs. It is implemented using C++17 constructs and it allows the definition of streaming programs by adopting the data-flow graph model, as extensively discussed in Fais [51]. Additionally, its provided abstractions are built on top of the building blocks offered by the FastFlow [55] C++ library. Thanks to this, WindFlow is able to offer a low-latency programming environment for streaming applications.

In conclusion, on one hand it is important to know the level of abstraction of the exposed interface of a given DaSP framework because this determines its *expressive power*. On the other hand, both the runtime and the mechanisms used to implement the provided abstractions characterize the achievable *performance*. Therefore, the choice of the right DaSP framework for the particular use case must always consider aspects such as the target system and the strictness of the imposed constraints on throughput and latency performance parameters [53].

## 4. The Processing Framework

**Figure 4:**
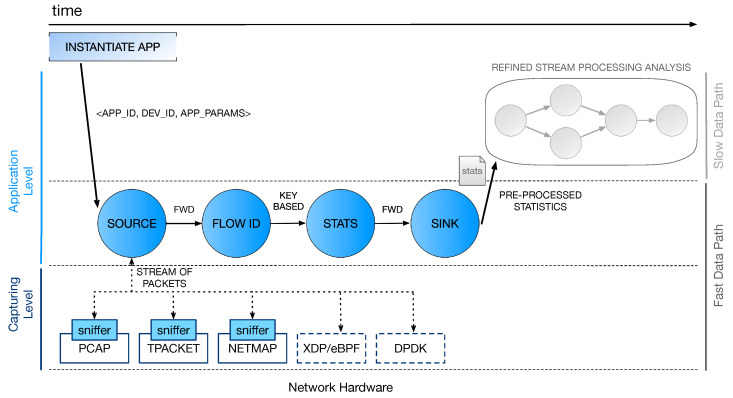
Processing pipeline of the example application. At the capturing level, three software packet-capturing tools are currently supported: pcap, TPACKET, and Netmap.

This work extends the prior work in Fais et al. [3], where we discussed the main perspectives and challenges in the adoption of Data Stream Processing in softwarized networks. In particular, we are designing a higher-level framework (see Section 2.3) that aims at improving the performance of packet-level analysis applications by taking advantage of suitable SPSs in conjunction with fast packet capturing tools. While our previous work did not provide an actual implementation, here the concept of a *multi-layer stream processing model* is applied to a first example application whose general structure is depicted in Figure 4 and will be covered in the next Section 5.

In this section, we give an overview of the framework architecture. Three main logical processing layers can be identified: the capturing layer, the first processing stage, and a second (optional) stage of computation that may implement more refined and aggregated statistics based on the results computed in the first stage.

### 4.1. Capturing Layer

The portion of processing at the *capturing level* constitutes the lower layer of the architecture. On one hand, this stage is in charge of the interaction with the NIC for collecting packets that must be analyzed by the application. On the other hand, it interacts with the source entity of the processing graph implementing the level above, feeding it with new packets from the network. The first task is implemented by using software solutions for high-performance packet capturing, such as the ones described in Section 2.2. As for the interaction with the layer above, this is defined through proper sniffer objects (see Figure 4) that are specifically designed for this purpose. In the example application, we selected Netmap to implement this first fast packet-capturing layer in software. More details on the sniffer implementation and the interaction with the source will be provided in Section 5, where we will also discuss some major optimizations designed to improve the application performance and keep up with the incoming packet rate from the capturing layer.

### 4.2. First Stream Processing Stage

This is the first processing stage and it works on a per-packet basis to possibly implement very complex analytical tasks. Monitoring computations are implemented with a suitable SPS and modeled as graphs of operators that elaborate and exchange tuples. In the example application, we chose WindFlow to implement the processing in this stage. The source is the most critical part of the graph. It is in charge of (*i*) interacting with the underlying capturing level component and (*ii*) creating tuples from the received packets, according to the tuple format of the SPS in use. Once the tuple is created, the packet can be released and the processing is applied to tuples as they traverse the whole graph (up to the sink).

### 4.3. Refined Stream Processing Analysis

This last stage of computation can implement processing tasks to be executed in the slow data path. An overview of the state of the network can be offered if we first gather information (i.e., join together streams of statistics) from several network devices. A refined analysis of these data can be useful for raising alerts upon anomaly detection or providing a visual summary of the collected statistics—for example, by generating a GUI that simplifies the network programmer work.

## 5. Example Application

To perform some preliminary experiments and show that the proposed design can be used for implementing per-packet analysis tasks, an example application has been implemented. Packets are processed one-at-a-time, UDP and TCP network flows are identified, and some statistics (e.g., packet counters, byte counters) are extracted. The first two levels of processing (capturing layer and first stream processing stage) have been implemented. Figure 4 describes the general structure of the application. From a high-level perspective, the processing is implemented by a linear sequence (pipeline) of operators. However, each computation stage can be replicated when necessary (assuming there are resources available to do so). For this reason, the graph of the running application can be more generic.

### 5.1. Structure and Implementation

By definition, a graph is always composed by at least one source and one sink. The source is the entry point in the graph. Three main tasks are delegated to this operator: (i) implement the logic required to capture packets from the network; (ii) create well-formed tuples starting form the packet content, according to the format required by the SPS in use; (iii) forward each tuple to the successive operator in the graph. The source entity has been implemented by using the Source abstraction exposed by WindFlow. The general structure of the implementation is the one in Listing 1. The business logic is implemented in the operator method of the class defining the source node. An *ad hoc* object called Sniffer provides the necessary interaction with the capturing layer to receive packets. Listing 2 shows its general structure. As the name suggests, the sniffer is used to *sniff* traffic from a given network interface to which is attached with the bind method. Packets are captured one by one by invoking the sniff_next method. Underlying this, Netmap methods are invoked to efficiently implement the packet capturing in the software. Each packet is parsed upon arrival and its content is used to initialize a new WindFlow tuple, then the packet is released. Each tuple is sent to the successive node in the graph at the end of the operator method. The execution of this method is triggered each time a new incoming packet is available in input (i.e., sniff_next returns a new valid packet). For each replica of the source, a single input stream is received from the underlying capturing stage and handled.

**Listing 1:**
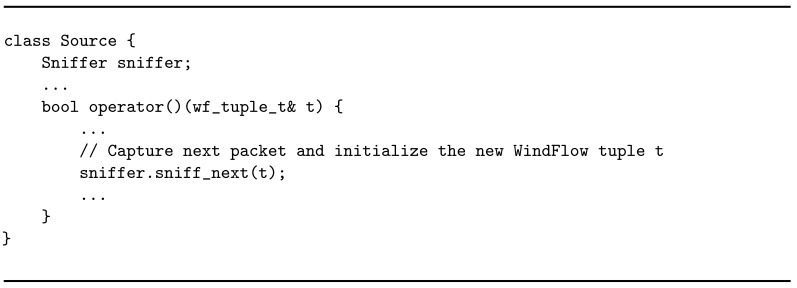
Business logic of the source operator.

**Listing 2:**
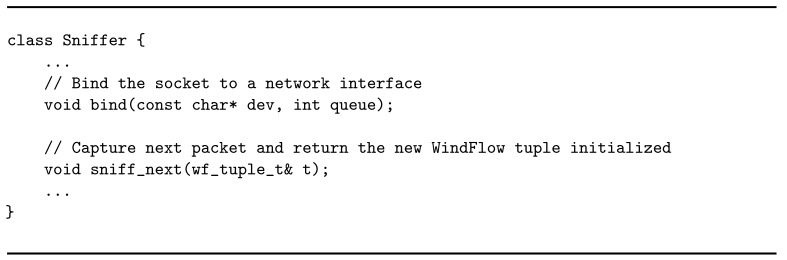
Structure of a sniffer object.

At this point, it is worth clarifying the distinction between packets and tuples. With the term *packet*, we identify network packets as they are captured from the NIC. A *tuple*, instead, is a flow item inside the streaming application processing graph (between source and sink). The tuple format is specified by the DaSP framework used, and this is true for all high-level stream processing systems for Big Data (e.g., Storm, Flink, WindFlow). Thus, we can see tuples as packet abstractions at the application processing level. A packet flow is typically defined by the 5-tuple IP source and destination addresses, source and destination ports, and transport protocol, and this definition is always true during the entire processing chain. The identification of logical tuple sub-streams, based on a selected key (or subset of keys) among the key-value pairs defining the tuples, is only significant between the current operator and its key-based successor. Indeed, this is something relevant for the tuple distribution strategy between adjacent operators only. For state-of-the-art DaSP frameworks (e.g., Storm, Flink), the adoption of key-based tuple distributions is the only way to support the replication of a stateful operator. Thus, each replica receives a subset of the tuples only and can keep an internal state for that sub-stream which is local to the replica itself. On one hand, this avoids the cumbersome implementation and management of a state shared among many parallel entities. On the other hand, if we are able to select a good key (characterized by a uniform distribution of values), the workload will be well-balanced across all the replicas.

Following the order in the graph, the second operator is flowid. Its main task is to compute a *flow identification number (ID)* for each tuple received in input. Therefore, it performs a *map* operation: for each incoming tuple, one tuple annotated with its corresponding *flow ID* is produced in output. The implementation uses the Map abstraction from the WindFlow API. As shown in Listing 3, the logic is defined in the operator method of the class defining the flowid node. The *flow ID* value is treated as *key* field for the tuples. This plays an active role in the distribution of the tuples towards the next operator in the graph, which is stats.

**Listing 3:**
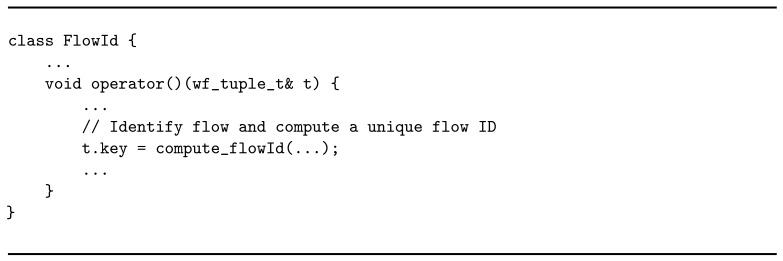
Business logic of the flowid operator.

The stats operator works on keys. The distribution of tuples between flowid and stats is key-based, meaning that the stream is logically partitioned on a certain key field—in this case, the *flow id* value. If stats is replicated, tuples belonging to the same traffic flow (identified by the quintuplet <src IP, dst IP, src port, dst port, protocol>) are handled by the same replica. This simplifies also the management of the internal state, which remains local to the replica rather than being shared among many entities (introducing overheads for the synchronization). Listing 4 gives an idea of the operator’s business logic. stats is a stateful entity which maintains a variety of per-flow statistics updated every time a new tuple is received with that *flow id*. An internal map data structure is populated by associating each flow ID with two types of counters: the first one tracks the number of packets per flow, while the second one counts the total amount of bytes carried by the flow. Moreover, the operator maintains a set of general statistics on the absolute number of different flows identified since the beginning of the analysis, as well as the total amount of processed packets. In addition, stats updates per-protocol counters (e.g., UDP, TCP) in terms of packet volumes and unique flow cardinality. The operator has been implemented as a WindFlow Map, and upon state update it annotates every processed tuple with its current position within the flow before sending it out. Notice that, since the key-based distribution guarantees flow consistency, the tuple position corresponds to the actual packet position in the flow.

**Listing 4:**
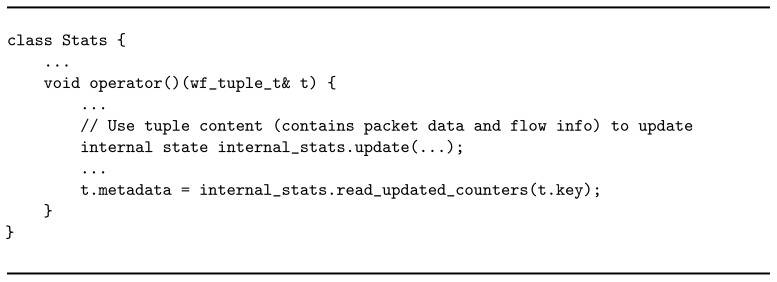
Business logic of the stats operator.

Last but not least, the sink operator terminates the tuple stream, which is the last stage in the processing graph. An idea of its internal logic is given in Listing 5. The tuple argument received in the operator main method is an optional type, used to manage the *end of stream* case. This is directly offered by the Sink WindFlow operator. When we receive the first non-valid tuple, this is interpreted as a signal that the stream is ended. At this point, the aggregated statistics evaluated in the operator can be exported on a file or may be visualized in some way for easier analysis. In the first case, there is room for the further processing of the produced analytics, and this can be a possible entry point for the second stage of processing in the slow data path.

**Listing 5:**
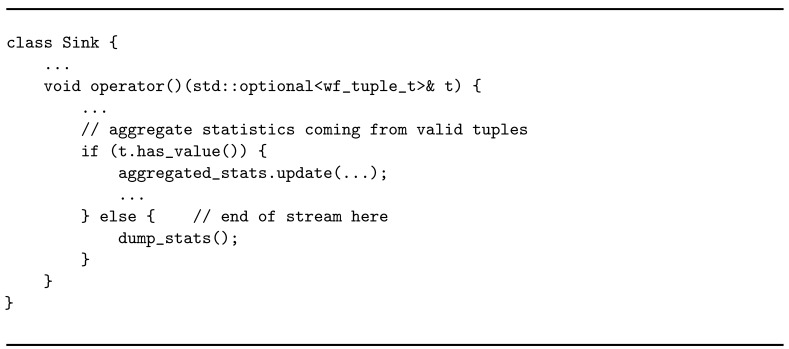
Business logic of the sink operator.

Once each operator has been defined with its business logic, parallelism degree, and type of tuple distribution from its predecessor, the application graph can be created by composing operators together. WindFlow offers specific constructs (PipeGraph, MultiPipe) for this purpose, which make it easy for the programmer to specify the application topology. Listing 6 gives an idea of the implementation of the application graph.

The framework offers two ways of inserting operators into the graph: add and chain. When an operator is *added* to the MultiPipe, it executes on a dedicated thread of the CPU. Moreover, its communication with adjacent operators is managed through Single Producer Single Consumer (SPSC) lock-less queues shared between threads. When the *chaining* feature is used, the current operator is combined together with the previous one. Chained operators execute on the same thread, and data forwarding is performed by using function or method calls rather than push and pop on a shared SPSC queue. However, *chaining* is feasible only if (*i*) the operators involved have the same parallelism degree, and (*ii*) the tuple distribution between the two operators is not key-based.

From the programmer perspective, enabling the chaining feature is as simple as using the chain method instead of the add method of the MultiPipe. In Listing 6, operators following the source are added in order to the MultiPipe construct. In accordance with the two conditions to apply chaining, flowid can be chained to the source and sink to stats if the involved operators are defined with the same number of replicas. However, stats can never be chained to flowid due to the partition of the stream by key.

In the end, once the graph is defined, the application is ready to be executed.

**Listing 6:**
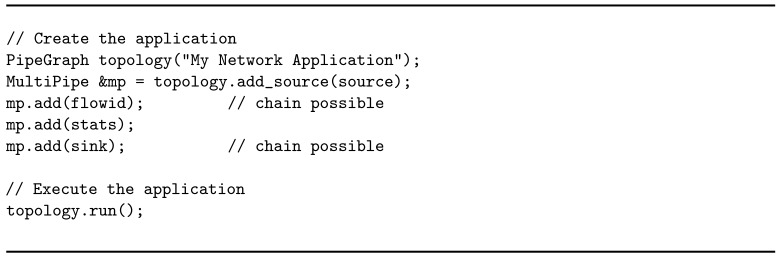
Construction of the application graph.

### 5.2. Optimizations

One of the aspects that emerge from the above description of the application structure is the expressive power and high abstraction level offered by SPSs—in this case, WindFlow. However, expressiveness necessarily comes at a cost in terms of performance. With this work, we would like to provide a preliminary evaluation of this cost: (*i*) in the case of a C++-based streaming library targeting multicores; (*ii*) for use cases in the networking domain, where performance requirements for implementing per-packet analysis at line rate are far more stringent than those for more generic applications in the big-data analysis domain.

In order to improve the performance achieved by the proposed example application, optimizations such as *chaining* and *memory pool* have been applied.

**Chaining** is an optimization feature offered by WindFlow, as already mentioned above. It is particularly beneficial in the case of very fine-grained computations, where having chained operators running on the same thread could lead to a better exploitation of the cores of the machine instead of having some of them underutilized. In the experimental evaluation (Section 6), the possible chaining configurations for the example application described above have been tested. We labeled as no-chain the standard version of the application and chain the version where chaining has been enabled.

**Memory pool** is a major optimization that has been implemented to improve the performance of the application. In the first version of the application implementation (standard WindFlow, no chaining), we identified a performance bottleneck related to the behavior of the default malloc implementation in the GNU C library (libc) in multi-threaded environments. In processing graphs defined with WindFlow, tuples are allocated in the source thread, and in the end they are freed in the sink thread. These two different threads (no chaining enabled) try to access to the same memory area and contend for acquiring the corresponding lock. Thus, this greatly limits the throughput the application is able to achieve in processing packets.

Our solution is based on two concepts: (i) The implementation of a logical communication channel between the sink and the source. The idea is that, in order to avoid the cost related to the lock, all malloc and free operations for each tuple must be executed by the same thread. (ii) The idea of having a pool of pre-allocated tuples. As long as there are tuples available in the pool, each new operation picks one of them instead of calling malloc. Conversely, as long as there is space available in the pool, each delete operation simply inserts the pointer of the tuple into the pool so that it can be recycled. Therefore, only a small number of malloc and free is called, when the pool is empty or full, respectively. Their cost is overall amortized in this way.

For the implementation, the Improved FastForward version of the Single Producer Single Consumer (SPSC) queues designed in [56] has been used. This communication channel between sink and source can be seen as a way of wrapping up the processing pipeline on itself. In practice, this is almost like having a circular flow of tuples: (i) tuples end their life cycle in the sink, after they’ve been processed, but if there is space in the channel/pool they are re-initialized and inserted there; (ii) at this point, they can be re-used by the source, that takes empty tuples from the pool and initializes them with the content of some newly received packets. In the evaluation (Section 6), we labeled as no-pool the version of the first implementation, based solely on WindFlow abstractions and pool the version where the channel/pool optimization is enabled.

In addition to using *chaining* and *memory pool*, a set of low-level micro optimizations has been extensively applied in the code to further improve the packet processing capacity. Such optimizations include socket parameter tuning (e.g., sync frequency for reading the packet ring), the extensive use of power-of-two buffer sizes so as to avoid the latency introduced by divisions, the adoption of suitable strategies to maximize the positive impact of data prefetching and cache reuse, and so on.

## 6. Experimental Evaluation

We present the preliminary results in terms of throughput obtained from the experimental evaluation of the example application presented in Section 5. Two machines are used to perform the experiments:

- The first one is equipped with a CPU Intel Core i7-3770K with 4 cores (8 hardware threads) and 4 GB of RAM. All cores dynamically share access to the last level cache L3 of 8 MB. Each core has a clock rate of 3.50 GHz and a L2 of 1 MB (L1 is 128 KB for data and 128 KB for instructions). The network interface used is an Intel Ethernet Controller XL710 for 40 Gb Ethernet QSFP+. This is the machine executing the traffic generation task.
- The second one has a CPU Intel Xeon E5-1660 v3 with 8 cores (16 hardware threads) and 64 GB of RAM. All cores dynamically share access to the last level cache L3 of 20 MB. Each core has a clock rate of 3.00 GHz and a L2 of 256 KB (L1 is 32 KB for data and 32 KB for instructions). Additionally, here the NIC is use is an Intel Ethernet Controller XL710 for 40 Gb Ethernet QSFP+. This is the machine that runs the streaming application. For reproducibility and performance, both frequency-scaling and sleep states deeper than C1 have been disabled.

The test environment has been configured with Netmap for both the generation phase on the first machine and the receiving phase on the second one. Moreover, the availability of FastFlow and WindFlow is required for running the example application.

Four versions of the tested application result from the possible combinations (presence/absence) of the two different optimization features presented in Section 5.2: *chaining* and *memory pool*.

### 6.1. Speed Test Configuration

**Figure 5:**
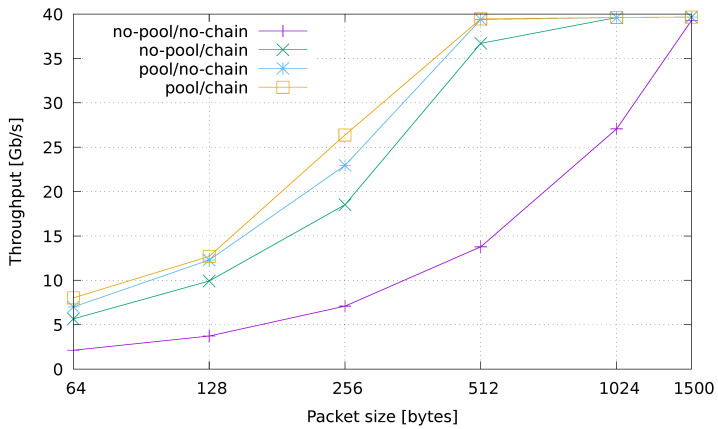
Speed test evaluation.

The first set of experiments are targeted at evaluating the overhead introduced by the streaming machinery in programming network applications such as the example one used here. In order to do this, we eliminated the internal processing logic of the application (the parsing of packets in the sniffer and general computation on tuples) by keeping a minimal configuration of the processing graph composed of a source and a sink only. Empty tuples are created in the source one for each packet received from the sniffer using Netmap. The tuples are sent to the sink, which simply counts them. The measurements taken for this minimal testing configuration are shown in Figure 5, which plots the achieved throughput as the size of the generated packets varies. The y-axis describes the throughput in Gb/s, while the x-axis reports the packet size in bytes. The scale of the x-axis is logarithmic.

In the basic configuration (no-pool/no-chain), the number of processed packets per second remains almost the same as the packet size changes. This is the case that allows us to perform a pure evaluation of how well a fully general-purpose streaming framework behaves when used for per-packet analysis purposes. The other three lines plotted in the figure describe the impact of selectively applying the optimizations (*pool*, *chaining*) to the basic no-pool/no-chain configuration. By looking at the plot, one can immediately notice that the two versions with the pool optimization enabled (pool/no-chain, pool/chain) are the ones performing better. Indeed, in both cases we are able to process packets at line rate with packet sizes of 512 bytes and larger, which is already a good estimate for a real-traffic scenario. The performance achieved by enabling chaining only (no-pool/chain) is much better than the baseline, and comes very close to the results obtained with the pool. This can be explained by the fact that chaining lets the source and sink operators run on the same thread, thus removing the performance degradation caused by lock contention in the malloc function.

### 6.2. Real-Traffic Configuration

In this test case, we aim at evaluating how the implemented application behaves when fed with real traffic. For this reason, we compare the results obtained by the applications in both the minimal and full-fledged configurations. In the latter, all of the application operators are enabled as well as the logic for every portion of the computation. The results are presented in terms of the number of analyzed packets per seconds and the portion of dropped packets with respect to the total amount of replayed packets. Real traffic is replayed from a pcap file at 10 Gb/s and 20 Gb/s. In all cases, the application is run four times for all possible combinations of the *pool* and *chaining* optimizations. Each test is replicated 10 times and the average throughput and drop-rate is reported. The standard deviation of all the measured quantities was always very low and, for this reason, is not reported in the plots.

The full-fledged application supports chaining between the first and second operators (source and flowid) and between the third and fourth ones (stats and sink). However, enabling the chaining of the first two operators was never beneficial in our experiments. This is easily explained by the fact that, in all the experiments, the bottleneck was always on the thread running the source operator. By chaining another operator, we add more variable workload on the same thread. This causes the upstream input network queue to fill up more often, forcing the network interface to drop more packets. For this reason, in the remaining of the section the *chain* optimization only refers to the chaining of the sink operator with its predecessor in the pipeline.

**Figure 6:**
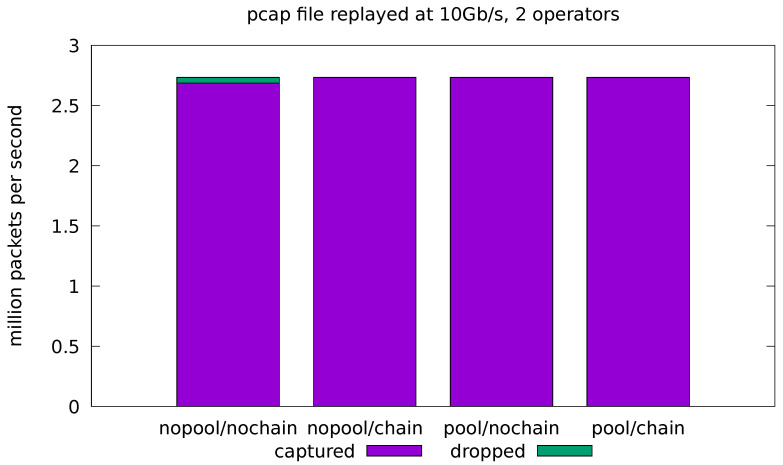
Evaluation on real-traffic replayed at 10 Gb/s from a pcap file. Application is run with source and sink operators only.

Figure 6 shows the results for the minimal application when processing the 10 Gb/s traffic. We can observe a significant drop rate in the base, non-optimized case, which becomes negligible when either of the two optimization is enabled. Note that a small number of dropped packets, in the order of a thousand or less dropped packets per second, is always observed in all of our tests. We attribute this to the non real-time nature of the Linux kernel. We took care of pinning all the application threads on CPU cores that were isolated from the Linux scheduler, but a longer list of careful configurations are needed to obtain a mostly jitter-free execution on Linux [57].

**Figure 7:**
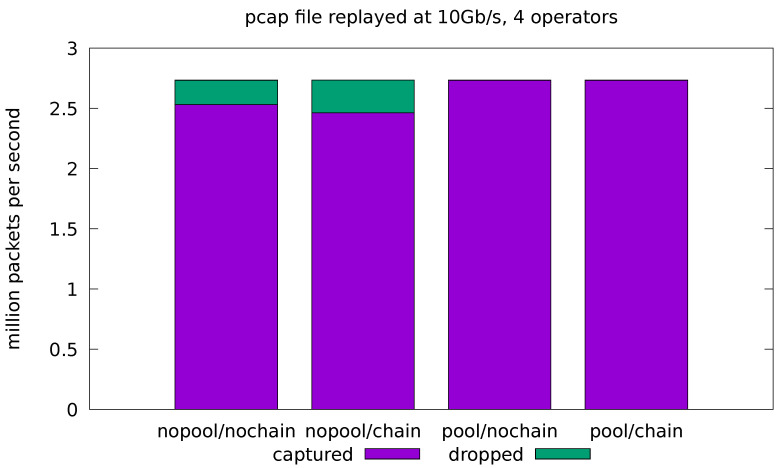
Evaluation on real-traffic replayed at 10 Gb/s from a pcap file. Application is run with all four operators in the processing pipeline.

**Figure 8:**
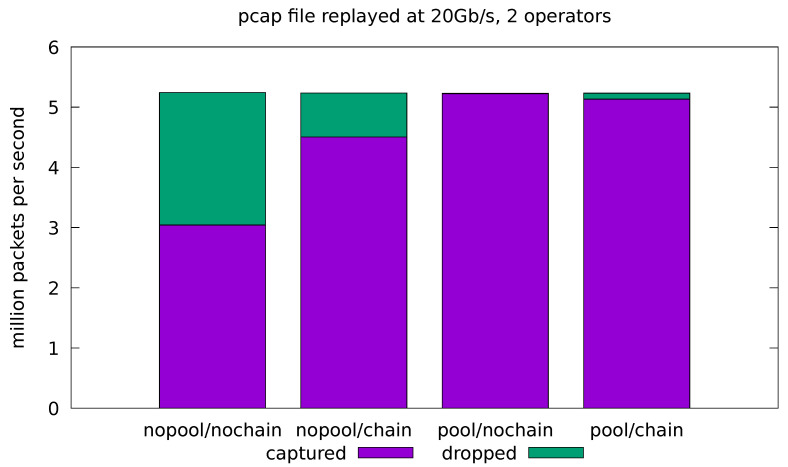
Evaluation on real-traffic replayed at 20 Gb/s from a pcap file. Application is run with source and sink operators only.

**Figure 9:**
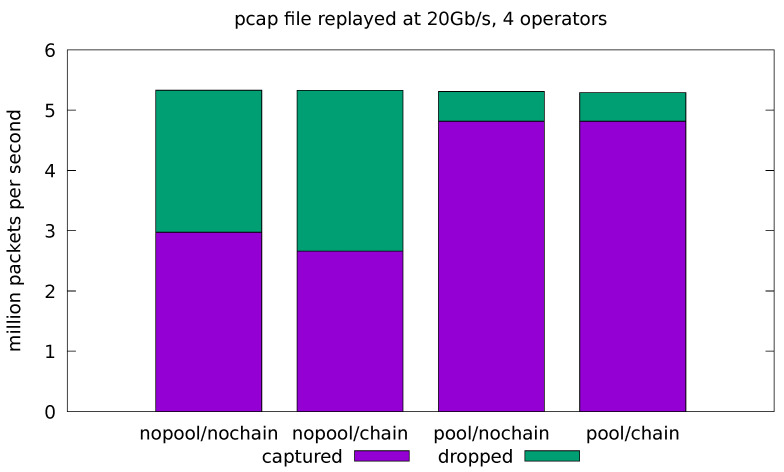
Evaluation on real-traffic replayed at 20 Gb/s from a pcap file. Application is run with all four operators in the processing pipeline.

Figure 7 shows the results for the full-fledged application processing the 10 Gb/s traffic. We can see that the *pool* optimization is essential to remove the cost of the malloc lock contention and reduce the drop rate to a negligible value. On the other hand, the *chain* optimization shows a small increase in the drop rate in the no-pool configurations. Note that, due to the limitations of chaining, the source and sink operators are still running in separate threads for this application, even with chaining enabled. This means that the lock contention on memory allocation is still present, thus negating the most important effect of chaining in our experiments. In general, the chaining optimization can have both positive and negative effects on the throughput (see also Figure 8 and Figure 9). These effects are caused by complex interactions in the caching subsystem and are hard to predict.

Overall, the 10 Gb/s experiments show that the framework is adequate for processing realistic traffic at 10 Gb/s speed, even when only one source thread is used, provided that the pool optimization is enabled. On the other hand, the application designer should always experiment with the different combinations of the chaining optimization, instead of settling on a default. The framework makes this experimentation simple, since enabling or disabling chaining is just a matter of calling a different method when composing the flow graph.

In the remaining experiments, we try to push the system to its limits by running the pcap replayer at its maximum speed, which is just short of 20 Gb/s in our scenario. Figure 8 and Figure 9 show the results for the minimal and full-fledged application, respectively. We can see that the base system (with all the optimizations turned off) is overwhelmed by the incoming stream of packets and the network interface is forced to drop almost half of them in both the minimal and full-fledged configurations. The chaining optimization is only helpful in the minimal configuration (Figure 8), where the application is turned into a single-threaded one and the malloc lock contention is avoided. At these rates, however, even the cost of the single-threaded malloc is too high, and the minimal application is able to cope with the incoming traffic only when the *pool* optimization is turned on. Enabling both *pool* and chain gives slightly worse throughput than just *pool* alone, since it causes the sink operator to interfere with the already almost saturated source.

Figure 9 shows that, in the most demanding 20 Gb/s, full-fledged configuration scenario, the *pool* optimization greatly reduces the amount of dropped packets, but a significant drop-rate is still visible. Note that, with respect to Figure 8, this scenario only involves two additional threads, running on separate CPU cores. The increased drop rate, therefore, cannot be explained by the additional computational costs, but most likely by the increased contention on the cache and memory subsystems. Further experimentation is needed to pinpoint the cause of the performance degradation in this scenario.

## 7. Conclusions and Future Directions

The paper presents a first attempt to apply the principles of Data Stream Processing (DaSP) to the design and development of network applications addressing packet-level analysis. As widely discussed in the article, current mainstream DaSP frameworks target distributed environments and are based on the Java Virtual Machine. Therefore, they are far from meeting the performance target required by the traffic speed of modern networks. Starting from [3], our approach began with the selection of WindFlow (based on C++ and targeting multi-core machines) as a viable candidate to work on in order to combine reasonable performance and powerful expressiveness.

With this starting point, the whole system has been conceived according to a two-tier processing scheme, in which the lower level has been improved in two main ways. On one hand, this is achieved by integrating software accelerated capture sources (such as Netmap). On the other hand, this is achieved by introducing few performance optimizations that specifically apply to the networking domain. As a result, the first processing tier of the system is able to handle and elaborate traffic data in real-time over the fast data path (i.e., on the arriving packets, directly). The output of the first processing layer can then be either directly accessed by the user or forwarded to a second stage for aggregation/elaboration/visualization. These latter operations are optional and much less critical in terms of performance, as they generally do not need the whole packet stream.

An example monitoring application has been implemented according to the described scheme and tested with artificial and real traffic patterns at 10 and 20 Gb/s traffic speeds. The application is composed of four operators, performing processing over the fast data path. As expected, the experimental results show that the DaSP system without optimizations do not meet the performance requirements of network applications running on the data plane. This is even more evident as the computation burden of the application increases. However, the applied optimization mechanisms proved to be beneficial and allowed the application to reach a packet throughput of nearly 20 Gb/s with a single processing pipeline on real input traffic.

These are indeed very promising results that open the way to new directions for future work in this area. In terms of performance, a set of additional optimizations can be added in order to better adapt the underlying framework to the typical conditions and data speed of network applications.

In terms of functionalities, a proper API to interact with standard controllers (e.g., ONOS) is still lacking and will be included in a follow-up version of the system.

## Abbreviations

The following abbreviations are used in this manuscript:

API	Application Programming Interface
BDSP	Big Data Stream Processing
COTS	Commodity Off The Shelf
CPU	Central Processing Unit
DaSP	Data Stream Processing
DPDK	Data Plane Development Kit
DPI	Deep Packet Inspection
eBPF	extended Berkeley Packet Filter
FPGA	Field Programmable Gate Array
GPU	Graphic Processing Unit
GUI	Graphical User Interface
INT	In-Band Network Telemetry
JVM	Java Virtual Machine
NFV	Network Function Virtualization
NIC	Network Interface Card
OS	Operating System
POF	Protocol Oblivious Forwarding
QoS	Quality of Service
SDN	Software Defined Network
SIEM	Security Information and Event Management
SmartNIC	Smart Network Interface Card
SPSC	Single Producer Single Consumer
XDP	eXpress Data Path
XFSM	eXtended Finite State Machines
